# Unsuccessful Weaning From Mechanical Ventilation in a Patient With An Immune-Mediated Necrotizing Myopathy: A Case Report That Demonstrates the Usefulness of Shear-Wave Elastography

**DOI:** 10.1016/j.chest.2024.01.002

**Published:** 2024-06-07

**Authors:** Ivo Neto Silva, Aileen Kharat, Florian Marzano, Elisa Marchi, José Alberto Duarte, Karim Bendjelid

**Affiliations:** a Department of Acute Medicine, Intensive Care Division, Geneva University Hospitals, Geneva, Switzerland; b Care Directorate, Geneva University Hospitals, Geneva, Switzerland; c Geneva Hemodynamic Research Group, Faculty of Medicine, University of Geneva, Geneva, Switzerland; d Centre of Physical Activity, Health and Leisure (CIAFEL), Faculty of Sport, University of Porto, Porto, Portugal; e Medicine Department, Division of Respiratory Medicine, Geneva University Hospitals, Geneva, Switzerland; f TOXRUN-Toxicology Research Unit, University Institute of Health Sciences-CESPU, Gandra, Portugal

A 39-year-old woman with a 3-year medical history of anti-signal recognition particle-positive immune-mediated necrotizing myopathy (IMNM) was admitted to the ED for acute respiratory distress. Four days prior to admission, creatine kinase levels were elevated (Fig 1). In the ED, arterial blood gas levels confirmed a severe respiratory acidosis (pH 7.11; Paco_2_ 12.9 kPa); the patient was intubated immediately for mechanical ventilation. The patient CT-scan workup showed multiple bilateral consolidation (right upper, middle, and left lower lobe) that was compatible with community-acquired pneumonia.

**Figure 1:**
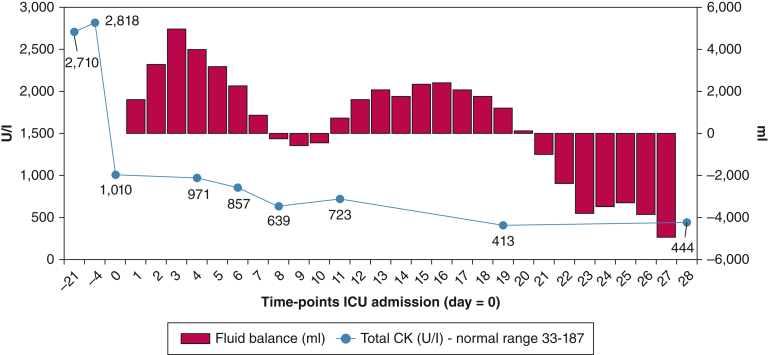
CK and cumulative fluid balance evolution during the hospital stay. CK = creatine kinase.

Controlled mechanical ventilation was performed during the first days (Fig 2) with a cumulative fluid balance of approximately +4,000 mL (Fig 1). A good collaborative state at day 4 allowed the assessment of the Medical Research Council Sum-Score that revealed no limb weakness (53/60 points), which was maintained during the entire ICU stay (Fig 2).

**Figure 2:**
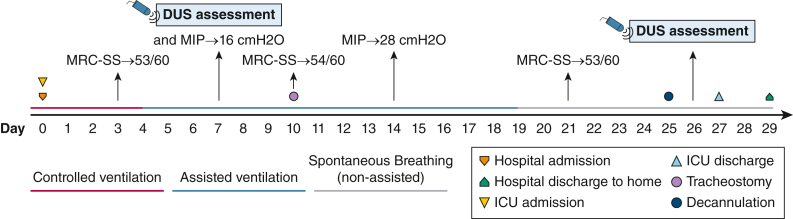
Hospital timeline. DUS = diaphragm ultrasound scan; MIP = maximal inspiratory pressure; MRC-SS = Medical Research Council Sum-Score.

Over the following days, several attempts failed to decrease pressure support (PS) on the ventilator, and the patient showed rapid signs of respiratory fatigue. A bilateral diaphragm ultrasound (DUS) assessment was performed with the use of conventional B-mode ultrasound scanning and shear-wave elastography (SWE) technique. DUS was performed with the use of a standardized technical approach.^1^ All the assessments were performed with mechanical ventilation support under two different conditions: high-PS (18 cmH_2_O) and low-PS (8 cmH_2_O).

The patient underwent tracheostomy on the day 10 and received rituximab (day 11) in the context of the IMNM. From day 19, she was weaned totally from any ventilatory support. On day 25, she underwent successful decannulation. One day after decannulation, a new DUS was performed (day 26: second time point). After day 27 in the ICU, the patient was transferred to the ward at room air. She was discharged 2 days later without any need for noninvasive respiratory support.

*Question 1:* Based on visualization of conventional ultrasound assessment of the left (Video 1, clips 1 and 3) and right (Video 2, clips 1 and 3) diaphragms, is it possible to observe structural changes?

*Question 2:* When the previous observations are put in the context of the related data (Fig 3), which physiologic and functional changes can be appreciated?

*Question 3:* After an SWE assessment of both diaphragms was performed, which biomechanical (stiffness) findings can be added to the clinical reasoning (Video 1, clips 2 and 4; Video 2, clips 2 and 4) (Fig 3)?

**Figure 3:**
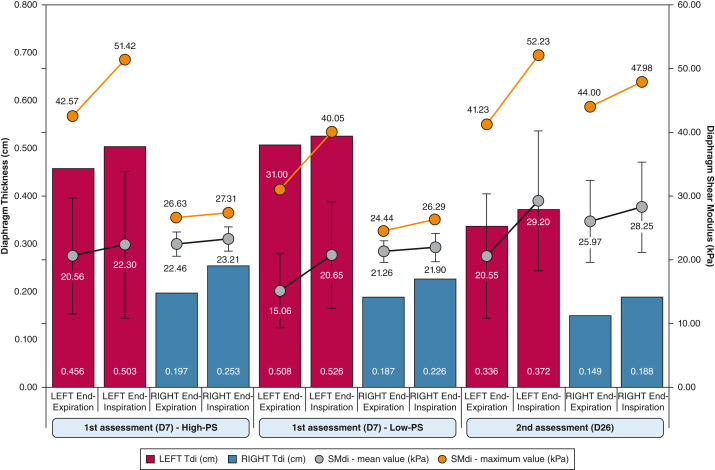
Evolution of left and right Tdi and shear modulus levels at different conditions and time points of assessment. Red and blue bars indicate the thickness of the left and right diaphragm, respectively. SMdi mean value and SD are represented by the gray plots; shear modulus maximum values are represented by orange plots. High-PS = 18 cmH_2_O of PS; Low-PS = 8 cmH_2_O of PS; PS = pressure support; SMdi = diaphragm shear modulus; Tdi = diaphragm thickness.

*Answer 1:* At day 7, B-mode ultrasound scanning showed a very hyperechogenic left diaphragm with some nonorganized hypoechogenic regions (Video 1, clip 1; Fig 4A), in what seems to be related to myofibrillar injury, probably because of the necrotizing process of underlying known disease. These injury signs showed great attenuation at the end of the ICU stay (second time point) (Video 3, clip 1). Both pleural and peritoneal membranes were thickened as well. The right diaphragm had a homogenous appearance despite higher echogenicity appearance than normal (Video 2; clip 2).

**Figure 4:**
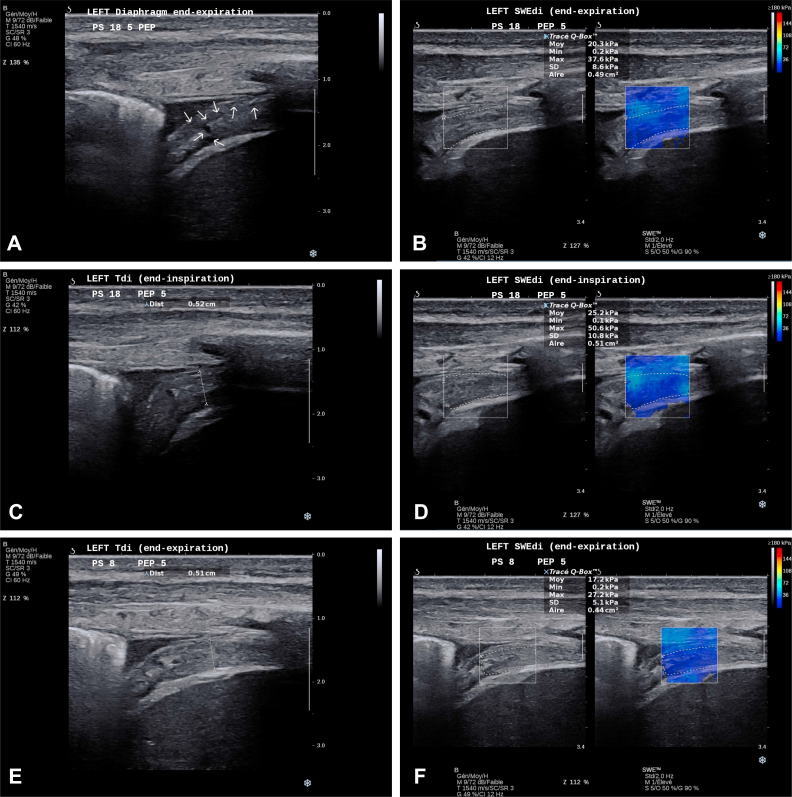

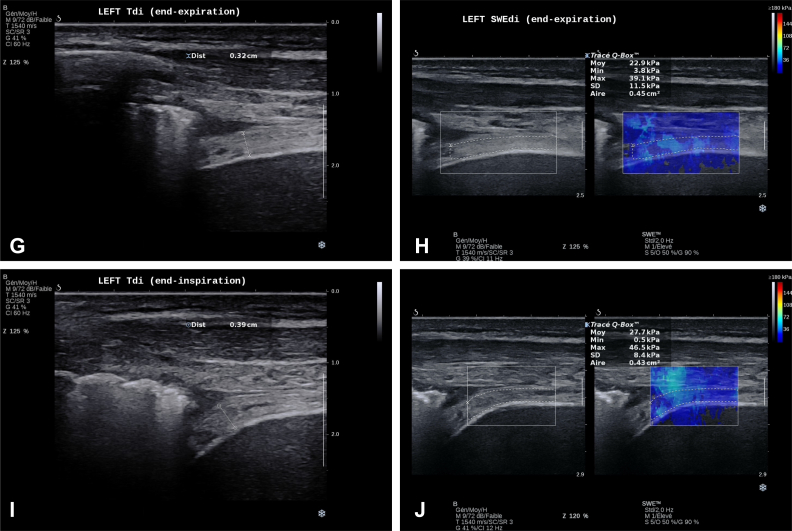
A-J, Left diaphragm at both time points of assessment. A, Left Tdi at end-expiration during high-PS (18 cmH_2_O): first time point of DUS assessment. White arrows indicate regions of diaphragmatic tissue injury. B, Left SWEdi at end-expiration during high-PS (18 cmH_2_O): first time point of DUS assessment. C, Left Tdi at end-inspiration during high PS (18 cmH_2_O): first time point of DUS assessment. D, Left SWEdi at end-inspiration during high-PS (18 cmH_2_O): first time point of DUS assessment. E, Left Tdi at end-expiration during low-PS (8 cmH_2_O): first time point of DUS assessment. F, Left SWEdi at end-expiration during low-PS (8 cmH_2_O): first time point of DUS assessment. G, Left Tdi at end-expiration: second time point of DUS assessment. H, Left SWEdi at end-expiration: second time point of DUS assessment. I, Left dTdi at end-inspiration: second time point of DUS assessment. J, Left SWEdi at end-inspiration: second time point of DUS assessment. Aire = area; Dist = distance; DUS = diaphragm ultrasound scan; Max = maximum; Min = minimum; Moy = mean; PEP = positive expiratory pressure; PS = pressure support; SWEdi = diaphragm shear-wave elastography; Tdi = diaphragm thickness.

*Answer 2:* With the B-mode ultrasound scan, we were able to objectify a very thick left diaphragm (example in Fig 4) when compared with the right side (example in Fig 5) with a diaphragm thickness (Tdi) of 0.508 cm vs 0.196 cm, respectively. In terms of motion, the left diaphragm presented a hypokinesis that was revealed by a small “hinge-like movement” with a hypoechogenicity-based fulcrum that was ventilation-dependent and that was more pronounced at high-PS (Video 1, clips 1 and 3). This passive behavior was consistent with a low diaphragm thickness fraction (TFdi) at both low-PS (3.54%) and high-PS (10.29%). The right diaphragm showed slightly better contractility (TFdi, 20.76% at low-PS and 28.81% at high-PS) despite presenting some ventilator contribution to the increase in TFdi (“pushing effect, explained by a higher TFdi at high-PS) and the presence of a delayed intercostal musculature activity.

**Figure 5:**
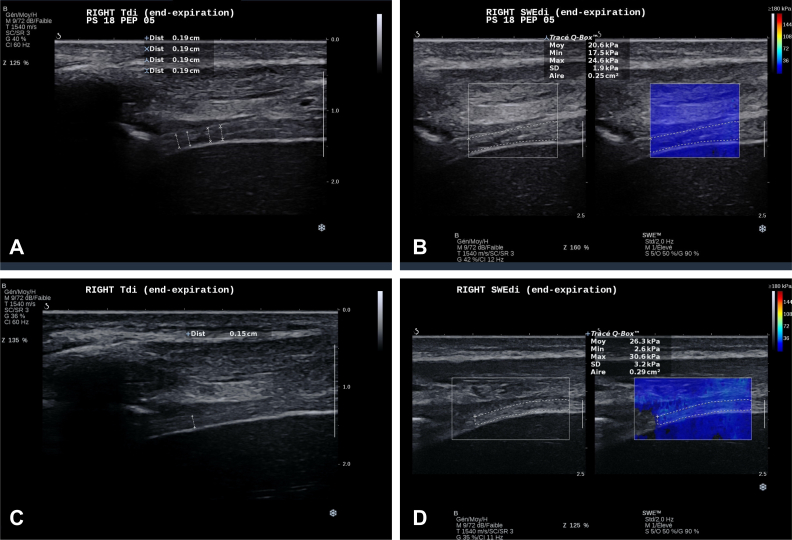
A-D, Right diaphragm at both time points of assessment. A, Right Tdi at end-expiration during high-PS (18 cmH_2_O): first time point of DUS assessment. B, Example right SWEdi at end-expiration during high-PS (18 cmH_2_O): first time point of DUS assessment. C, Right Tdi at end-expiration: second time point of DUS assessment. D, Example right SWEdi at end-expiration: second time point of DUS assessment. Aire = area; Dist = distance; DUS = diaphragm ultrasound scan; Max = maximum; Min = minimum; Moy = mean; PEP = positive expiratory pressure; PS = pressure support; SWEdi = diaphragm shear-wave elastography; Tdi = diaphragm thickness.

DUS performed at ICU discharge (day 26) (Videos 3 and 4, clip 1) showed an end-expiration Tdi decreased in both diaphragms (difference day 7 to day 26: left Tdi, −0.172 cm; right Tdi, −0.048 cm); still, TFdi maintained almost the same values (Fig 3). After the previous findings, the left diaphragm showed a lesser degree of dysfunctional inspiratory movement.

*Answer 3:* At the first time point (day 7), the left and right diaphragm showed an overall condition that was quite similar to diaphragm shear modulus (SMdi) mean values, except the lower SMdi mean value (Fig 3) at end-expiration during low-PS (15.06 kPa). Compared with the right diaphragm, the left diaphragm showed at least twice more SMdi maximum measured values (Fig 3) and SD mean values (Fig 3); both values showed a trend to higher values during the inspiration. This may indicate a greater variability in left diaphragm tissue stiffness, possibly linked with greater muscle tissue injury and fibrosis, as seen in previous classic B-mode assessment.

At ICU discharge, SWE for the left diaphragm showed an increased SMdi mean value (+6.9 kPa) at end-inspiration, but the remaining SMdi maximum values and SD mean values remained constant. This could be considered as an improvement in left diaphragm contractility in a still high heterogenous “ground” (muscle tissue).

On the right side, a simultaneous and sustained overall increase in all SMdi markers was observed: during both end-expiration and end-inspiration moments (Fig 3). The previous may indicate higher basal muscle stiffness and better inspiratory contractility, despite a similar TFdi to day 7. Clinically, this improvement was observed by a progressive better inspiratory function (maximal inspiratory pressure; day 7: 16 cmH_2_O; day 14: 28 cmH_2_O) and successful weaning and decannulation (Fig 2)

## Discussion

IMNM is one of the subgroups of the idiopathic inflammatory myopathies, a rare group of autoimmune diseases, also known as myositis.^2^^,^^3^ The overall impact of IMNM is quite well-described in the literature^3^^,^^4^ and includes limb weakness with a proximal and lower limb predominance^3^ and high levels of creatine kinase, which correlates well with the amount of necrotic muscle fibers.^3^^,^^5^^,^^6^ However, only a few cases regarding the impact of the disease on the respiratory muscles, acute respiratory failure episodes,^7^, ^8^, ^9^ and the need of invasive mechanical ventilation have been reported.^10^^,^^11^

In ICU patients, diaphragm dysfunction is almost twofold more prevalent than limb weakness at the time of mechanical ventilation liberation and is associated with higher rates of weaning failure^12^ and higher mortality rates.^12^^,^^13^ Diaphragm assessment through classic ultrasound technology has been used to assist clinical decisions related to the weaning process and identification of diaphragm dysfunction.^14^^,^^15^ More recently, the addition of ultrasound SWE to the assessment of the diaphragm has been proposed and shown to be reliable.^16^

SWE provides real-time estimation of muscle mechanical properties (ie, stiffness) based on Hooke’s law. Through different techniques, it uses a push beam (“acoustic force”) to generate shear waves. This generates tissue displacement that is identified by the ultrasound pulse. At first, a measurement of shear wave velocity (m·s^−1^) is obtained that is then used to calculate shear modulus that is retrieved in kilopascals.^17^ This measure varies according to tissue properties and is displayed on a B-mode view, in the form of a color map (elastogram) within a preselected region of interest; a greater level of shear modulus indicates a “stiffer” tissue. A comprehensive review on the principles and biomechanical concepts of the SWE application in the assessment of the skeletal muscle can be found elsewhere.^17^ Regarding its use for the diaphragm assessment, changes in SMdi values seem to reflect changes in the transdiaphragmatic pressure during isovolumetric inspiratory efforts in healthy patients^18^ or in patients who receive mechanical ventilation.^19^ It may be a promising tool for treatment guidance in patients with diaphragmatic dysfunction.^20^ At present, SWE is not yet a widespread technique, because it is available only on some advanced equipment from certain manufacturers. However, this does not mean that it requires any additional technical expertise for experienced sonographers. Specific attention should be paid to avoid tissue compression. Usual probe can be used for this technique.

The authors presented a rare case of a patient with anti-signal recognition particle-positive IMNM with a prolonged weaning process caused by diaphragmatic dysfunction without limb muscle weakness. This case highlights the usefulness of both conventional and advanced DUS techniques for the clinical decision-making process in patients who are difficult to wean.

Using classic DUS assessment, we were able first to describe some nonorganized hypoechogenic regions (Video 1, clip 1) that may be related to the myofibrillar injury in the left diaphragm. This may be due to the underlying necrotizing process of the disease. Consequently, left Tdi was increased greatly (more than two times) when compared with the contralateral side, which could be explained by a greater extent of the damage therefore greater thickness.^21^ This difference could be related partly to positive fluid balance and the presence of edema caused by higher capillary leak and tissue permeability.

When assessing diaphragm biomechanical properties assessment (SWE) in this context of muscle injury, we observed inferior mean SMdi values in the left diaphragm when compared with the right side at both high-PS and low-PS, which corroborates the very few data available for patients who are mechanically ventilated: an increase in Tdi is associated with a reduction in SMdi values and is directly related to the myotrauma concept.^21^^,^^22^ Additionally, we analyzed the SMdi maximum and SD mean values obtained within the assessed region of interest and observed at least three times higher values for both variables. We hypothesized that the muscular tissue heterogeneity is due to muscle injury or infiltration.^22^ Another interesting point to be raised is that the patient presented higher values of SMdi at end-expiration compared with those described for critically ill patients.^16^ This is due to preexisting muscle endomysial fibrosis from the IMNM chronic condition.^3^

After successful weaning and decannulation (timeline: Fig 2), the DUS assessment showed a left Tdi much thinner than in the first time points of the DUS assessment. Additionally, we observed a right Tdi reduction as well, which raises explanations on the role of edema and fluid balance (Fig 1). Interestingly, although right end-expiratory SMdi increased when it was compared with the first assessment according to previously mentioned rationale of the inverse relation between Tdi and SMdi, the left SMdi had the same level during the first assessment, which possibly showed tissue incapacity to develop further spontaneous basal muscle tension. Other values of tissue heterogeneity, such as mean SMdi maximum values and mean SD values remained at the same levels.

The baseline structural changes seem to have impacted the left diaphragm mobility and contractility and reproduced a dysfunctional “hinge-like movement” (Video 1, clip 1). This was associated with a TFdi value of 10.59%, which is below the accepted but not consensual cutoff for extubation success criteria of approximately 30% (Fig 3).^14^^,^^23^ Interestingly, we were able to contextualize TFdi concerning contractility of both diaphragms. TFdi fell below the previously described values when PS was reduced to 8 cmH_2_O, which indicates that both diaphragms presented extremely low contractile activity and that their low thickness during inspiration could be understood by a “smashing effect” from both thoracic and abdominal compartments that combined to a pushing effect from the positive pressure ventilation. This rationale can be supported by the relatively low mean values of SMdi difference between inspiration and expiration for both sides, with the exception of those values observed during low-PS that is influenced largely by the low end-expiratory value of SMdi and contrasts with the expected increases in SMdi during progressive inspiratory loading that are seen in healthy subjects (Fig 3).^18^

The authors reported a rare case of anti-SRP-positive IMNM that presented a prolonged weaning process because of diaphragmatic dysfunction. This case highlights the potential of diaphragm ultrasound scanning to evaluate diaphragm tissue injury possibly related to the underlying necrotizing process of the disease, the importance of this assessment for the weaning-related clinical workup, and the added value of the novel application of SWE for diaphragm mechanical properties assessment and its participation to the rationale for tissue injury. This last point may open a new horizon to a more comprehensive diaphragm dysfunction assessment in patients who are critically ill and myopathic.

## Reverberations

1. *D* *US can be a valuable tool during the mechanical ventilation weaning process in critical care patients. It adds great value when sequential assessment is performed under different clinical conditions, allowing to deem muscle tissue response and contractility.*
2. *Even though quite a rare clinical scenario, anti-* *signal recognition particle* *-positive IMNM may present difficult weaning caused by specific diaphragm dysfunction. In these situations of a high-complexity muscle dysfunction (ICU-known sources of “aggression” and the disease itself), a bilateral DUS assessment should be proposed.*
3. *In patients with anti-* *signal recognition particle* *-positive IMNM who present with diaphragm impairment, tissue injury may be observed with a conventional B-mode ultrasound scanning approach.*
4. *Diaphragm assessment with SWE is a novel technique. It brings valuable information to tissue assessment and therefore to clinical reasoning, notably in what concerns the determinants of biomechanical properties such as edema/infiltration and fibrosis.*

## Financial/Nonfinancial Disclosures

None declared.

## Supplementary Data

Video 1 B-mode and shear-wave elastography assessment of LEFT diaphragm at the first time point were performed under mechanical ventilation support with two different conditions: high-PS (clips 1 and 2) and low-PS (clips 3 and 4) for the same positive expiratory pressure (5 cmH_2_O). Technically, the 10-MHz to 2-MHz linear transducer was placed longitudinally, between the 8th and 10th intercostal space in the mid-axillary or anterior-axillary line. Diaphragm is the tissue between two hyperechogenic layers: the parietal pleura as the nearest line and the peritoneum as the deeper one. All SWE assessments were performed with maximally available frequency of acquisition (ie, 2 Hz). White arrows indicate nonorganized hypoechogenic regions, possibly related to a myofibrillar injury.

Video 2 B-mode and shear-wave elastography assessment of RIGHT diaphragm at the first time point were performed under mechanical ventilation support with two different conditions: high-PS (clips 1 and 2) and low-PS (clips 3 and 4), for the same positive expiratory pressure (5 cmH_2_O). Technical approach was performed as described for Video 1.

Video 3 B-mode and shear-wave elastography assessment of LEFT diaphragm at the second time point (clips 1 and 2). Technical approach was performed as described for Video 1.

Video 4 B-mode and shear-wave elastography assessment of RIGHT diaphragm at the second time point (clips 1 and 2). Technical approach was performed as described for Video 1.
